# Mutagenicity of New Lead Compounds to Treat Sickle Cell Disease Symptoms in a *Salmonella*/Microsome Assay

**DOI:** 10.3390/ijms11020779

**Published:** 2010-02-25

**Authors:** Jean Leandro dos Santos, Eliana A. Varanda, Lídia Moreira Lima, Chung Man Chin

**Affiliations:** 1 Lapdesf–Laboratório de Pesquisa e Desenvolvimento de Fármacos, Departamento de Fármacos e Medicamentos, Faculdade de Ciências Farmacêuticas, Univ Estadual Paulista–UNESP, Rodovia Araraquara Jaú Km. 01, 14801-902, Araraquara, SP, Brazil; 2 Departamento de Ciências Biológicas, Faculdade de Ciências Farmacêuticas, Univ Estadual Paulista–UNESP, Rodovia Araraquara Jaú Km. 01, 14801-902, Araraquara, SP, Brazil; 3 LASSBio–Laboratório de Avaliação e Síntese de Substâncias Bioativas, Faculdade de Farmácia, Univ Federal do Rio de Janeiro–UFRJ, Centro de Ciências da Saúde, Cidade Universitária, Ilha do Fundão, 21.944-190–Rio de Janeiro, RJ, Brazil

**Keywords:** AMES test, mutagenicity, sickle cell, phthalimide derivatives

## Abstract

A series of phthalimide derivatives planned as drugs candidates to treat the symptoms of sickle cell anemia were evaluated in a mutagenicity test using strains of *Salmonella typhimurium* TA100 and TA102, without and with addition of S9 mixture, with the aim to identify the best structural requirements for a drug candidate without genotoxic activity. The compounds (1,3-dioxo-1,3-dihydro-2*H*-isoindol-2-yl)methyl nitrate (**1**); (1,3-dioxo-1,3-dihydro-2*H*-isoindol-2-yl)ethyl nitrate (**2**); 3-(1,3-dioxo-1,3-dihydro-2*H*-iso-indol-2-yl)benzyl nitrate (**3**); 4-(1,3-dioxo-1,3-dihydro-2*H*-isoindol-2-yl)-*N*-hydroxy-benzenesulfonamide (**4**); 4-(1,3-dioxo-1,3-dihydro-2*H*-isoindol-2-yl)benzyl nitrate (**5**) and 2-[4-(1,3-dioxo-1,3-dihydro-2*H*-isoindol-2-yl)phenyl]ethyl nitrate (**6**) presented mutagenic potency ranging between 0–4,803 revertants/μmol. These results allowed us to propose that a methyl spacer linked to a nitrate ester subunit associated to *meta* aromatic substitution decreases mutagenicity.

## 1. Introduction

Sickle cell disease is a hematological genetic disease that results from a single point mutation of *β*Glu6 in Hb to *β*Val6 in HbS. In the deoxygenated state there is an interaction between the mutation region of one HbS molecule and a region defined by *β*Phe85 and *β*Leu88 in the heme pocket of another HbS. This interaction leads to polymerization that causes the normally flexible red blood cells (RBC) to adopt rigid, sickle like shapes that block small capillaries initiating the vaso-oclusive process, and causing local tissue damage and severe pain [1,2].

Hydroxyurea (HU) has been utilized for at least two decades in the treatment of sickle cell disease. The beneficial effect of HU has been associated with its capacity to induce fetal hemoglobin synthesis. Fetal hemoglobin (HbF) is predominant during fetal life and presents greater affinity for oxygen that normal hemoglobin. HbF concentration decreases after birth but there is an association between increasing this kind of hemoglobin with reduction of painful episodes in patients with sickle cell disease [3]. Besides, HU is metabolized to nitric oxide that has an important role in the vaso-oclusive process. HU is known as a ribonucleotide reductase inhibitor and as an antineoplasic drug that provokes DNA alterations (genotoxic activity), inducing gene and chromosome mutations [4,5].

As a result, the introduction of new drugs with the same beneficial effect for treatment of the sickle cell disease without genotoxic activity is necessary and urgent. Exploring the ability of HU, as a NO-source, we have designed the compounds (1,3-dioxo-1,3-dihydro-2*H*-isoindol-2-yl)methyl nitrate (**1**); (1,3-dioxo-1,3-dihydro-2*H*-isoindol-2-yl)ethyl nitrate (**2**); 3-(1,3-dioxo-1,3-dihydro-2*H*-isoindol-2-yl)-benzyl nitrate (**3**); 4-(1,3-dioxo-1,3-dihydro-2*H*-isoindol-2-yl)-*N*-hydroxybenzenesulfonamide (**4**); 4-(1,3-dioxo-1,3-dihydro-2*H*-isoindol-2-yl)benzyl nitrate (**5**) and 2-[4-(1,3-dioxo-1,3-dihydro-2*H*-iso-indol-2-yl)phenyl]ethyl nitrate (**6**) as potential NO-donors. These compounds **1–6** have presented an analgesic and anti-inflammatory profile and the capacity to stimulate the gamma globin RNAm synthesis using K562 cells [6].

The generation of reactive species could be responsible for a mutagenic activity of several compounds with NO-donor properties. It has been reported that the capacity to generate nitric oxide by some compounds in the AMES test is responsible for observed mutagenic activity due to nitrosation of amines by NOx [7,8]. The nitric oxide induces mutations in *Salmonella typhimurium* strains used in the AMES test and can damage the DNA and lead to mutations of the DNA base sequence [9].

*In vitro* mutagenicity studies are an important part of discovery activities and are frequently critical in determining the future of a drug candidate. A drug candidate that is active in a mutagenicity test or that produce mutagenic metabolites by activation in a microsomal enzyme system generally will be discarded in favor of a backup candidate [10].

The objective of this study was to evaluate which compounds presented mutagenic activity in the AMES assay and to trace the structure-mutagenicity relationship profile in order to identify potentially hazardous phthalimide drug candidates. Genetic toxicology testing in drug discovery and optimization serve to quickly identify mutagens and remove them from development. In this work, the mutagenicity activities of compounds were evaluated in AMES tester *Salmonella typhimurium* strains TA100 and TA102 that are capable of detecting mutations that cause substitution of base pairs. Results from genetic toxicology tests, in combination with an adequate pharmacology profile are used as the basis to approve clinical trials of drug candidates.

## 2. Results and Discussion

Table 1 shows the number of revertants/plate, the standard deviation and the mutagenic index (MI) after the treatments with the compounds, in the two different strains of *Salmonella typhimurium*, with or without metabolic activation. Table 2 shows the mutagenic potencies (rev/μmol) of compounds observed using *Salmonella typhimurium* TA100 and TA102 in presence (+S9) and absence (−S9) of metabolic activation.

Compounds **1**–**6** were designed using hybridization as a molecular modification strategy and they were selected based on reports about tumor necrosis factor alpha inhibition and considering structure-activity relationships of phthalimide derivatives [6]. Compounds **1**, **2**, **3**, **5** and **6** are nitrate organic ester derivatives and the compound **4** is a sulphonylhydroxamic acid derivative.

HU exhibited mutagenicity in strains TA100 and TA102, in the presence and the absence of metabolic activation. In TA102 strain, the concentration of 468 μmol/plate induced a reduction in number of revertants, consequence of the toxicity caused by HU. The mutagenicity was observed at 234 μmol/plate with mutagenic index of 2.0 and 2.2 in presence and absence of metabolic activation, respectively (Table 1). The mutagenic potency were 1.7 revertants/μmol (+S9) and 2.0 revertants/μmol (−S9) to TA102 strain (Table 2). Using TA100, mutagenic index higher than 2 was observed at concentrations above 234 μmol/plate. The mutagenic potency was 1.8 revertants/μmol and 1.1 revertants/μmol in the presence and absence of metabolic activation for TA100, respectively. These results indicate that HU has a mutagenic potential at high concentrations above 234 μmol/plate using TA100 e TA102 *Salmonella* strain, although a previous study had showed the absence of mutagenicity of HU tested up 0.5 mg per plate in TA100, TA98 and TA1537 [11]. There is no increase of HU mutagenicity in metabolic activation condition when compared with S9 absence (−S9) in prokaryotic cells. In eukaryotic cell, a study of HU, in mammalian (V79) cells, reported microsomal activation-dependent mutagenicity and found that the addition of catalase inhibited microsome-mediated mutagenicity, indicating that hydrogen peroxide was involved in the formation of mutagenic DNA lesion [12].

In general, the mutagenicity of compounds **1–6** was observed mainly in the presence of metabolic activation (Table 1). The capacity to generate nitric oxide and/or radical species by nitrate ester seems to be dependent of the presence cystein residue. This condition occurs in the presence of metabolic activation (+S9) and could be a possible explication for this event.

Compound **1** exhibited a mutagenic index of 2.4 in the TA100 strain in the absence of metabolic activation (0.112 μmol/plate) and 2.3 in the TA102 strain in the presence of metabolic activation (0.056 μmol/plate). The mutagenic potency found was 1,795 revertants/μmol and 4,803 revertants/μmol, respectively, for TA100 (−S9) and TA102 (+S9).

Compound **2** shows mutagenicity using strain TA100 at all the tested concentrations in the presence of metabolic activation. The higher value of the mutagenic index was 4.0 at 0.085 μmol/plate. Mutagenicity was observed at 0.085 μmol/plate in TA100 in the absence of metabolic activation (−S9). The mutagenic potency observed in TA100 was, respectively, 1,041 revertants/μmol and 4,025 revertants/μmol in the presence and absence of metabolic activation.

Compound **3** showed no mutagenicity at the used concentrations, although the test with TA102 strain in the presence of metabolic activation and the concentration of 3.58 μmol/plate provided a mutagenic index of 1.9, showing signals of mutagenic activity.

Compound **4** only exhibited mutagenic activity at 15.7 μmol/plate in the TA100 strain with metabolic activation (MI = 2.7). The mutagenic potency observed in compound **4** in the presence of metabolic activation using TA100 was 13.6 revertants/μmol.

Compound 5 at 3.58 μmol/plate showed in TA100 strain (+S9) a mutagenic index of 2.0 and a mutagenic potency of 31 revertants/μmol, while in the absence of metabolic activation (−S9), at the same concentration it only showed evidence of mutagenicity (MI = 1.9). The concentration of 1.8 μmol/plate in the absence of S9 in strain TA102, showed evidence of mutagenicity with MI equal to 1.9.

Compound **6**, the phenyl-bridged derivative of compound **2**, exhibited in the TA100 strain, in the presence of metabolic activation, mutagenic activity in all concentrations. The higher mutagenic index in a non toxic concentration was 5.8 at 2 μmol/plate. The mutagenic potency observed in compound **6** using metabolic activation in TA100 was 1,393 revertants/μmol.

Compounds **1**, **2**, **3**, **5** and **6** are phthalimide derivatives containing a nitrate ester subunit as NO-donor group. Several reports in the literature have demonstrated that NO induces mutagenesis using the *Salmonella* mutagenicity assay, specifically in tester strains that detect base-pair substitution mutations at the *hisG46* mutation (e.g., TA100 and TA1535). Interestingly, NO was not mutagenic in TA102 and TA104 developed to detected oxidative mutagens at the base-pair substitution mutation *hisG428* [13,14].

Compounds **1** and **2** are alkylphthalimide derivatives. Comparing compounds **1** and **2**, we observed higher mutagenic index values in the latter. Table 2 shows that in TA100 strain (−S9) the mutagenic potency of compound 2 was 4,025 revertants/μmol, while compound 2 presented a mutagenic potency of 1,795 revertants/μmol.

Comparing compound **2** to compounds **3** and **5**, it was observed that the former has higher mutagenic potency. The structural difference among these compounds is a substitution of methylene in compound **2** by a phenyl subunit present in compounds **3** and **5**. These data lead us to suggest that the aryl derivatives, *i.e.*, those which presented an aromatic ring linked to a phtalimide subunit (compounds **3** and **5**), showed lower mutagenicity than alkyl derivatives.

Compound **4** is a sulfohydroxamic derivative (Table 1), which does not present the nitrate ester subunit, common to all compounds that presented mutagenic potency of 13.6 revertants/μmol. It is reported in the literature that hydroxylamine derivatives, or derivatives of hydroxamic acid present mutagenicity due to the capacity to generate radical species after oxidation [15–17]. The possible mutagenicity of the compound **4**, which occurs only in the presence of metabolic activation, could be attributed to the formation of an oxidized derivative or a radical compound. Comparing the concentration of the compound **4** used in the test to the aryl derivatives (**3**, **5** and **6**) it is observed that although mutagenic activity appears, this is only observed in high concentrations.

Comparing compound **5** to its regioisomer (compound **3**), we observed a more discreet profile of mutagenicity of compound **5** in relation to the compound **3**. This observation could be confirmed by data of mutagenic potency, while the compound **5** presented a potency of 31 revertant/μmol, the compound **3** did not present mutagenic activity.

Comparing compound **6** to compound **5**, both with *para-* substitution, it is observed that the phenethyl spacer increases mutagenicity when compared to a benzyl spacer. This same fact is observed comparing compound **1** (methyl) and **2** (ethyl) and could be due to an adequate distance to form reactive species that interacts with DNA. This conclusion is based on NO-donor data that show no difference of NO release determining by Griess reaction between the compounds **1** and **2**.

According to Table 2, the mutagenicity of all compounds is more accentuated with alkyl phthlimides derivatives than aryl phthalimides derivatives. TA100 detected mutagenic activity for compounds **2**, **4**, **5** and **6** whereas TA102 only detected mutagenic activity for compound **1**.

Although some compounds of this study presented a mutagenic index higher than 2, it is important to note that drugs as metronidazole and nitrofurazone present mutagenic indexes higher than 14 [18]. So, other genotoxic studies using eukaryotic cells must be realized to investigate the capacity of the compounds **1–6** to cause genetic alterations.

## 3. Experimental Section

### 3.1. Chemicals

Hydroxyurea (HU, CAS No. 127-07-1), dimethylsulfoxide (DMSO, CAS No. 67-68-5), nicotinamide adenine dinucleotide phosphate sodium salt (CAS No. 11-84-16-3), d-glucose-6-phosphate disodium salt (CAS No. 3671-99-6), magnesium chloride (CAS No. 7786-30-3), l-histidine monohydrate (CAS No. 7048-02-4), d-biotin (CAS No. 58-85-5), sodium azide (CAS No. 26628-22-8), 2-anthramine (CAS No. 613-13-8) and 2-aminofluorene (CAS No. 153-78-6) were purchased from Sigma Chemical Co. (St. Louis, MO, USA). Oxoid Nutrient Broth No. 2 (Oxoid, England) and Difco Bacto Agar (Difco, USA) were used as bacterial media. d-Glucose (CAS No. 154-17-6), magnesium sulfate (CAS No. 7487-88-9), citric acid monohydrate (CAS No. 5949-29-1), potassium phosphate dibasic anhydrous (CAS No. 7758-11-4), sodium ammonium phosphate (CAS No. 13011-54-6), sodium phosphate monobasic (CAS No. 7558-80-7), sodium phosphate dibasic (CAS No. 7558-79-4), sodium chloride (CAS No. 7647-14-5) were purchased from Merck (Whitehouse Station, NJ, USA).

### 3.2. Preparation of Compounds **1–6**

The compounds were prepared through condensation of aminoalcohol derivatives with phthalic anhydride. The alcohol function of the phthalimide derivatives were converted to the final compounds containing nitrate ester functions [5]. Compound **4** was prepared by the previously described condensation of sulphonyl chloride [19] with hydroxylamine. The compounds **1–6** were characterized by ^1^H- and ^13^C-NMR, IR and MS. The purity was confirmed by elemental analysis and HPLC and in general was 99% for all compounds.

### 3.3. Metabolic Activation System (S9 Mixture)

The S9 fraction, prepared from livers of Sprague–Dawley rats treated with the polychlorinated biphenyl mixture Aroclor 1254, was purchased from Molecular Toxicology Inc. (Annapolis, MD, USA). The metabolic activation system consisted of 4% of S9 fraction, 1% of 0.4 M MgCl_2_, 1% of 1.65 M KCl, 0.5% of 1M d-glucose-6-phosphate disodium and 4% of 0.1 M b-nicotinamide adenine dinucleotide phosphate sodium in 0.1 M, 50% of 0.2M phosphate buffer and 39.5% of sterile distilled water [20].

### 3.4. Bacterial Strains

TA100 and TA102 strains of *Salmonella typhimurium* were kindly supplied by Dr Bruce N. Ames from The University of California, Berkeley, USA. For all assays, an inoculum (200 μL) of a thawed permanent culture was added to 20 mL of Oxoid Nutrient Broth No. 2 and incubated at 37 ºC with shaking until a concentration of approximately 1–2 × 10^9^ bacteria per milliliter was obtained.

### 3.5. Mutagenicity Assay

The Salmonella mutagenicity assay was performed by pre-incubating the test compounds for 20–30 min with *Salmonella typhimurium* strains TA100 and TA102, with and without metabolic activation (S9 mixture) [21]. Compound **1** was tested at the following concentrations: 0.007; 0.014; 0.028; 0.056; 0.112 μmol per plate. Compound **2** was used at 0.01; 0.021; 0.042; 0.085 and 0.170 μmol per plate. Compound **3** was used at 0.224; 0.488; 0.896; 1.8 and 3.58 μmol per plate. Compound **4** was used at 0.98; 1.96; 3.92; 7.85 and 15.7 μmol per plate. Compound **5** was used at 0.244; 0.488; 0.896; 1.796 and 3.584 μmol per plate. Compound **6** was used at 0.12; 0.25; 0.5; 1.0 and 2.0 μmol per plate.

These doses were determined after the toxicity tests had been carried out. In all subsequent assays, the upper limit of the dose range tested was either the highest non-toxic dose or the lowest toxic dose determined in this preliminary assay. Toxicity was apparent either as a reduction in the number of his ± revertants, or as an alteration in the auxotrophic background (*i.e.*, background lawn).

The various concentrations of compounds to be tested were added to 500 μL of buffer pH 7.4 and 100 μL of bacterial culture and then incubated at 37 ºC for 20–30 min. After this time 2 mL of top agar was added to the mixture and poured on to a plate containing minimum agar. The plates were incubated at 37 ºC for 48 h and the his ± revertant colonies were manually counted. The influence of metabolic activation was tested by adding 500 μL of S9 mixture (5%). All experiments were performed in triplicate.

The standard mutagens used as positive controls in experiments without S9 mix were sodium azide (1.25 μg/plate) for TA100 and daunomycin (3 μg/plate) for TA102. 2-Anthramine (1.25 μg/plate) was used with TA100 and 2-aminofluorene (1.25 μg/plate) with TA102 in the experiments with metabolic activation. DMSO served as the negative (solvent) control (100 μL/plate).

The statistical analysis was performed with the Salanal computer program, adopting the Bernstein model [22]. The mutagenic index (MI)—the average number of revertants per plate divided by the average number of revertants per plate from the negative (solvent) control—was also calculated for each dose. A sample was considered positive when the MI was equal to or greater than 2 for at least one of the tested doses and if it had a reproducible dose–response curve [23,24].

## 4. Conclusions

Phthalimide derivatives with an ethyl spacer linked to a nitrate ester subunit contribute to generate compounds with higher mutagenic properties when compared with a methyl spacer. Furthermore, the introduction of nitrate ester subunit in the *meta-* aromatic position seems to decrease mutagenicity. The results indicate the structural requirement of phthalimide derivatives through mutagenic activity relationship study for the development of a new drug candidate to treat sickle cell disease symptoms’ and allow us to characterize compound 3 as a new promising drug to treat this hematological disorder.

## Figures and Tables

**Table 1.** Mutagenic activity expressed as the mean and standard deviation of the number of revertants/plate in bacterial strains TA100 and TA102 exposed to compounds (1–6) and HU, at various doses, with (+S9) or without (−S9) metabolic activation.

		**Revertants/plate in *Salmonella typhimurium* strains**			

**Compounds**	**Concentration μmol/plate**	**TA100**		**TA102**	

		**+S9**	**−S9**	**+S9**	**−S9**

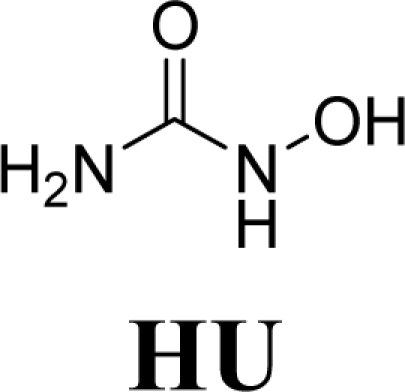	0	129.3 ± 8.1	143 ± 4.8	372.3 ± 20.5	370 ±17.2
	29.25	178.33 ± 15.1* (1.4)	149 ± 8.9 (1.0)	399 ± 31 (1.1)	411.3 ± 9.8 (1.1)
	58.5	240 ± 3** (1.8)	159 ± 11 (1.1)	484.6 ± 36 (1.3)	498 ± 15.3 (1.3)
	117	220.7 ± 17.8** (1.7)	268.7 ± 13.5* (1.9)	563.3 ± 22** (1.5)	602 ± 8.7* (1.6)
	234	276 ± 27.7** (2.1)	345 ± 21.2** (2.4)	764.3 ± 20** (2.0)	799.6 ± 12** (2.2)
	468	315.3 ± 16.1** (2.4)	358.6 ± 5.5** (2.5)	593 ± 23** (1.6)	550 ± 7.5** (1.5)

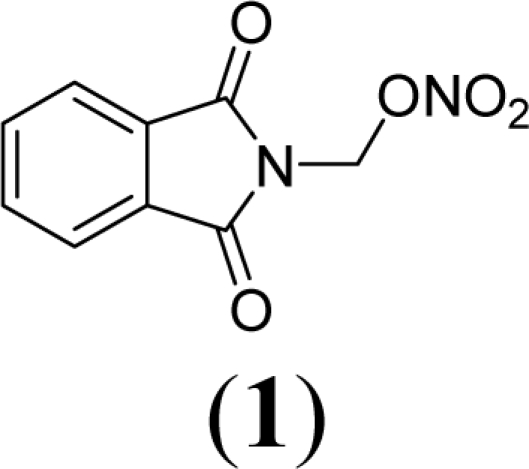	0	129.3 ± 8.1	136.7 ± 2.4	213.5 ± 15.5	197.33 ± 16.01
	0.007	126.3 ± 8.1 (1.0)	140.5 ± 17 (1.0)	288 ± 11.3 (1.3)	249.7 ± 12 (1.2)
	0.014	133.3 ± 11 (1.0)	161.7 ± 11 (1.2)	320.8 ± 17* (1.5)	263.7 ± 15 (1.3)
	0.028	140.3 ± 2 (1.1)	192.7 ± 3.06 (1.4)	385 ± 21.8** (1.8)	261.3 ± 20 (1.3)
	0.056	154 ± 14.2 (1.2)	234 ± 39.5** (1.7)	499 ± 8.9** (2.3)	153 ± 21 (0.8)
	0.112	155 ± 5.9 (1.2)	335 ± 15.7**(2.4)	351.2 ± 12*(1.6)	141 ± 18 (0.7)

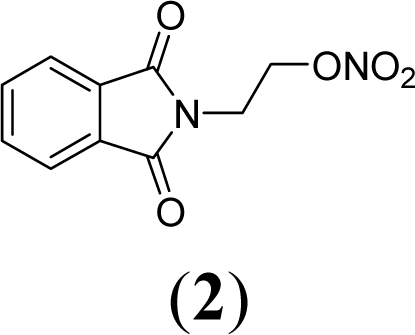	0	104 ± 7.4	115 ± 13.2	219 ± 8.9	323 ± 10.2
	0.01	335 ± 10.8**(3.2)	171 ± 38.9* (1.5)	249 ± 11.7 (1.1)	394 ± 30 (1.2)
	0.021	354 ± 6.9**(3.4)	200 ± 13.3* (1.7)	269 ± 10.6 (1.2)	411 ± 24 (1.3)
	0.042	397 ± 25.9** (3.8)	223 ± 15.04* (1.9)	239 ± 22.5 (1.1)	452 ± 35.4 (1.4)
	0.085	416 ± 15.2** (4.0)	226 ± 20.2* (2.0)	247 ± 21.4 (1.1)	468 ± 23 (1.4)
	0.170	261 ± 11** (2.5)	165 ± 11.1* (1.4)	205 ± 32 (0.9)	518 ± 14** (1.6)

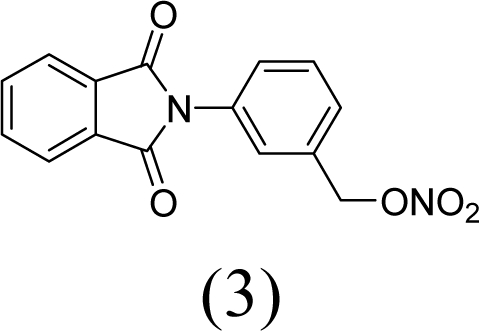	0	129 ± 8.1	179 ± 8.72	222 ± 12.5	254.7 ± 14.6
	0.224	146 ± 5 (1.1)	216.7 ± 10.2 (1.2)	272 ± 26 (1.2)	281 ± 25 (1.1)
	0.488	153 ± 13.1 (1.2)	234.5 ± 24.6 (1.3)	378 ± 6.1* (1.7)	286 ± 4.2 (1.1)
	0.896	163 ± 9.3(1.2)	266.3 ± 8.4* (1.5)	399.3 ± 12* (1.8)	303 ± 9.2 (1.2)
	1.8	165 ± 12.5 (1.3)	294.5 ± 5.3* (1.6)	405 ± 11* (1.8)	395.7 ± 9** (1.5)
	3.58	155 ± 8.2 (1.3)	323.7 ± 10.2* (1.8)	431 ±17.9* (1.9)	410.3 ± 9.7* (1.6)

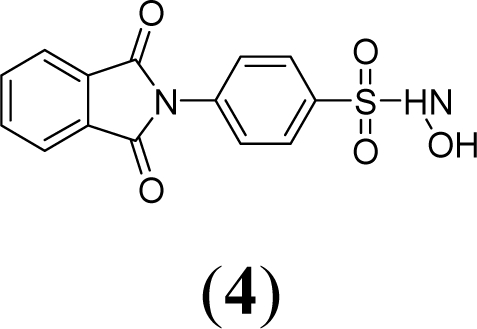	0	129.3 ± 8.14	126.7 ± 12.1	312.3 ± 27.5	197.3 ± 16.1
	0.98	160 ± 12.4 (1.2)	146.3 ± 5.1 (1.1)	323 ± 5.54 (1.0)	222 ± 12 (1.1)
	1.96	175.7 ± 2 (1.4)	172.7 ± 7.8 (1.4)	385 ± 33.1 (1.2)	241 ± 13 (1.2)
	3.92	194.2 ± 15 (1.5)	190 ± 11.3* (1.5)	423.7 ± 22 (1.3)	251 ± 19.2(1.3)
	7.85	206 ± 13.6** (1.6)	208 ± 12.5* (1.6)	443 ± 14.4 (1.4)	232 ± 13.1(1.2)
	15.7	354 ± 6.5* (2.7)	155 ± 2.8 (1.22)	295 ± 4.9 (0.9)	210 ± 17 (1.1)

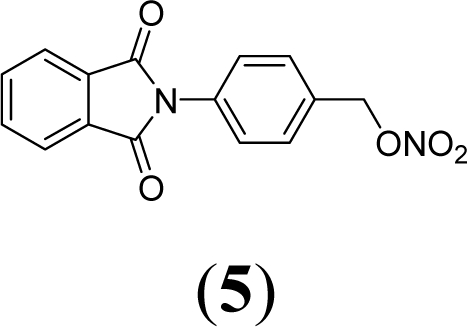	0	129.3 ± 8.1	179 ± 8.7	372.3 ± 27.5	260.1 ± 11.6
	0.224	157 ± 13 (1.2)	220 ± 18 (1.2)	385.7 ± 13 (1.0)	340.3 ± 9.7 (1.3)
	0.488	184.7 ± 16* (1.4)	239 ± 11.5 (1.3)	427.3 ± 5 (1.1)	373.5 ± 21 (1.4)
	0.896	192.7 ± 16* (1.5)	261 ± 10.2 (1.4)	436.7 ± 21 (1.2)	391.2 ± 6.3* (1.5)
	1.8	216.7 ± 18** (1.7)	303,8 ± 17* (1.7)	390.3 ± 9.3(1.0)	486.8 ± 20** (1.9)
	3.58	263 ± 12.3** (2.0)	349 ± 5.6** (1.9)	350 ± 6 (0.9)	420 ± 8.6* (1.6)

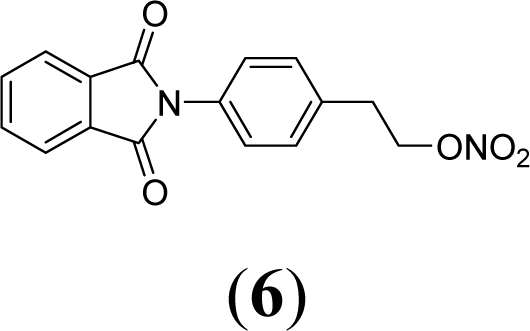	0	129.3 ± 8.1	116.7 ± 12.4	295.3 ± 28	197.3 ± 16.1
	0.12	363 ± 25.2** (2.8)	121.5 ± 14 (1.0)	335.3 ± 11 (1.1)	245 ± 6.9 (1.2)
	0.25	478.7 ± 4.22 **(3.7)	135.3 ± 12 (1.1)	372.3 ± 27.5 (1.3)	262 ± 19.1 (1.3)
	0.5	628 ± 40.8** (4.9)	138.3 ± 13 (1.2)	406 ± 14.1 (1.4)	286.7 ± 18 (1.4)
	1	739 ± 25.4** (5.7)	153.3 ± 5 (1.3)	465 ± 10.6* (1.6)	305.7 ± 10* (1.5)
	2	750 ± 13.6** (5.8)	177 ± 23.6* (1.5)	447 ± 19.8* (1.5)	201 ± 4.7 (1.0)

Positive control***	-	2545 ± 73.6	2231 ± 66.9	812 ± 26.1	881 ± 56.1

0 = negative control (DMSO–100 μL/plate)

* *P* < 0.01 or

** P < 0.05 (ANOVA). The values in parenthesis = mutagenic index. Numbers represent averages of triplicates from the three different experiments ± the standard deviation.

*** Positive control: sodium azide (1.25 μg/plate) for TA100 (−S9), daunomycin (3 μg/plate) for TA102 (−S9) and 2-anthramine (1.25 μg/plate) for TA100 (+S9) and 2-aminofluorene (1.25μg/plate) for TA102 (+S9).

**Table 2.** Mutagenic potencies (rev/μmol) observed for HU and compounds (1–6) with positive mutagenicity (MI ≥ 2) in TA100 and TA102 *Salmonella typhimurium* strains in presence (+S9) and absence (−S9) of metabolic activation.

**Compounds**	**TA100**		**TA102**	
	**(+S9)**	**(−S9)**	**(+S9)**	**(−S9)**
HU	1.8	1.1	1.7	2.0
1	-	1795	4803	-
2	1041	4025	-	-
3	-	-	-	-
4	13.6	-	-	-
5	31	-	-	-
6	1393	-	-	-

## References

[1] Steinberg MH Sickle cell anemia, the first molecular disease: Overview of molecular etiology, pathophysiology and therapeutic approaches ScientificWorldJournal 2008 25 1295 1324 19112541 10.1100/tsw.2008.157 PMC5848659

[2] Raghupathy R Billett HH Promising therapies in sickle cell disease Cardiovasc. Hematol. Dis. Drug Targets 2009 9 1 8 10.2174/187152909787581354 19275572

[3] Steinberg MH Pathophysiologically based drug treatment of sickle cell disease Trends Pharmacol. Sci 2008 27 204 210 16530854 10.1016/j.tips.2006.02.007

[4] Donehower RC An overview of the clinical experience with hydroxyurea Semin. Oncol 1992 19 11 19 1641651

[5] Friedrisch JR Prá D Maluf SW Bittar CM Mergener M Pollo T Kayser M da Silva MA Henriques JA da Rocha Silla LM DNA damage in blood leukocytes of individuals with sickle cell disease treated with hydroxyurea Mutat. Res 2008 649 213 220 17988936 10.1016/j.mrgentox.2007.09.005

[6] Santos JL Chung MC Lima LM Lanaro C Costa FF Use of Phthalimide and/or sulphonamide derivatives in the treatment of diseases which require reducing the TNF-alpha levels and exogenous source of nitric oxide, phthalimide derivatives, sulphonamide derivatives, and a method for obtaining a sulphonamide derivative PCT Int Appl WO2009073940 2009

[7] Abu-Shakra A The mutagenic activity of the S-nitrosoglutathione/glutathione system in *Salmonella typhimurium* TA1535 Mutat. Res 2003 539 203 206 12948829 10.1016/s1383-5718(03)00131-1

[8] Donovan PJ Smith GT Lawlor TE Cifone MA Murli H Keefer LK Quantification of diazeniumdiolate mutagenicity in four different *in vitro* assays Nitric Oxide 1997 1 158 166 9701054 10.1006/niox.1996.0109

[9] Arroyo PL Pigott VH Mower HF Cooney RV Mutagenicity of nitric oxide and its inhibition by antioxidants Mutat. Res 1992 281 193 202 1371842 10.1016/0165-7992(92)90008-6

[10] Xu JJ *In vitro* toxicology: Bringing the in silico and *in vitro* Comput. Toxicol 2007 1 21 32

[11] Bruce WR Heddle JA The mutagenic activity of 61 agents as determined by the micronucleus, *Salmonella* and sperm abnormality assays Can. J. Genet. Cytol 1979 21 319 334 393369 10.1139/g79-036

[12] Ziegler-Skilkakis K Schwarz LR Andrae U Microsome- and hepatocyte-mediated mutagenicity of hydroxyurea and related aliphatic hydroxamic acids in V79 Chinese hamster cells Mutat. Res 1985 152 225 231 4069149 10.1016/0027-5107(85)90065-x

[13] Tamir S Lewis RS Walker TR Deen WM Wishnok JS Tannenbaum SR The influency of delivery rate on the chemistry and biological effects of nitric oxide Chem. Res. Toxicol 1993 6 895 899 8117930 10.1021/tx00036a021

[14] Saliim ET Abu-Shakra A Effect of hydrogen peroxide on nitric oxide (NO)-induced mutagenicity in *Salmonella typhimurium* Teratog. Carcinog. Mutagen 2001 21 349 359 11746249 10.1002/tcm.1023

[15] Wei CI Cohen MD Swartz DD Fernando SY Corbett MD Mutagenicity of some monoaromatic hydroxamic acids Toxicol. Lett 1985 24 111 116 3883575 10.1016/0378-4274(85)90148-1

[16] Wang CY Mutagenicity of hydroxamic acid for *Salmonella typhimurium* Mutat. Res 1977 56 7 12 339075 10.1016/0027-5107(77)90235-4

[17] Wang CY Linsmaier-Bednar EM Lee MS King CM Mutagenicities of *N*-acyl-*N*-arylhydroxylamines for *Salmonella* Chem. Biol. Interact 1988 67 215 223 3056625 10.1016/0009-2797(88)90059-2

[18] Bosquesi PL Almeida AE Blau L Menegon RF Santos JL Chung MC Toxicity of nitrofuran drugs J. Bas. Appl. Pharm. Sci 2009 29 231 238

[19] Lima LM Castro P Machado AL Fraga CAM Lugnier C Moraes VLG Barreiro EJ Synthesis and anti-inflammatory activity of phthalimide derivatives, designed as new thalidomide analogs Bioorg. Med. Chem 2002 10 3067 3073 12110331 10.1016/s0968-0896(02)00152-9

[20] Garner RC Miller EC Miller JA Liver microsomal metabolism of aflatoxin B1 to a reactive derivative toxic to *Salmonella typhimurium* TA 1530 Cancer Res 1972 32 2058 2066 4404160

[21] Maron DN Ames BN Revised methods for the *Salmonella* mutagenicity test Mutat. Res 1983 113 173 215 6341825 10.1016/0165-1161(83)90010-9

[22] Bernstein L Kaldor J McCann J Pike MC An empirical approach to the statistical analysis of mutagenesis data from the *Salmonella* test Mutat. Res 1982 97 267 281 6750390 10.1016/0165-1161(82)90026-7

[23] Santos FV Colus IMS Silva MA Vilegas W Varanda EA Assessment of DNA damage induced by extracts and fractions of *Strychnos pseudoquina*, a Brazilian medicinal plant with antiulcerogenic activity Food Chem. Toxicol 2006 44 1585 1589 16730111 10.1016/j.fct.2006.03.012

[24] Varella SD Pozetti GL Vilegas W Varanda EA Mutagenic activity in waste from an aluminum products factory in *Salmonella*/microsome assay Toxicol.Vitro 2004 18 895 900 10.1016/j.tiv.2004.05.003 15465657

